# Auditor perspectives on barriers to achieving healthcare worker hand hygiene compliance goals

**DOI:** 10.1017/ash.2025.27

**Published:** 2025-02-12

**Authors:** Joseph Leszczynski, Kevin M. Gibas

**Affiliations:** 1 Brown University School of Public Health, Providence Rhode Island, USA; 2 Department of Medicine, Warren Alpert Medical School of Brown University, Providence Rhode Island, USA; 3 Department of Epidemiology & Infection Prevention, Rhode Island Hospital, Providence Rhode Island, USA

## Abstract

We surveyed hand hygiene auditors to identify barriers to healthcare worker hand hygiene compliance in our health system. Most auditors reported being comfortable providing staff feedback on hand hygiene practices; however, there was substantial variability in their comfort level based on the occupation of the staff member receiving feedback.

## Introduction

Hand hygiene is the most important practice in reducing the transmission of infections in healthcare settings.^1^ Despite extensive data and knowledge about the importance of healthcare worker (HCW) hand hygiene practices, healthcare institutions often struggle to consistently achieve high rates of HCW hand hygiene compliance.^2^ Low HCW hand hygiene compliance may contribute to increased rates of healthcare-associated infections, prolonged hospital length of stay, antimicrobial resistance, disability, and increased healthcare costs.^2^ These issues highlight the need for a better understanding of factors that influence HCW hand hygiene compliance and for innovative programs to improve compliance.^3^

As part of our hand hygiene improvement program, we examined barriers to achieving high HCW hand hygiene compliance from the perspective of hand hygiene auditors. Auditors monitor hand hygiene compliance and provide feedback to HCWs, a process requiring both technical skill and the ability to navigate hierarchical and interpersonal dynamics.^4^ We conducted a survey exploring auditor experiences with training, providing feedback to HCWs, and the use of technology for audit reporting. Insights from this survey highlight the importance of targeted interventions that address auditor education, accessibility and availability of hand hygiene infrastructure, and institutional culture around hand hygiene.

## Methods

As part of a quality improvement needs assessment, we conducted a survey of staff who perform audits to monitor HCW hand hygiene at Brown University Health’s (BUH). Auditors consist mainly of nursing, infection prevention, and administrative staff or volunteers. Approximately 70% of auditors report conducting audits ≥1 days per week. All auditors complete an online hand hygiene auditor training program developed by the Department of Epidemiology and Infection Prevention prior to being granted access to the platform to record hand hygiene audits.

The survey was voluntary and anonymous, and each participant could only complete the survey once. Staff who conducted audits between January and July 2024 were emailed a link and QR code to complete the survey. The survey was conducted between July 30, 2024 and August 14, 2024. The survey consisted of eight multiple choice questions and one free response question focusing on auditor experiences providing feedback to staff, the education/training auditors receive on conducting audits, auditor experience using the platform for recording hand hygiene audits, and staff perceptions of trends related to hand hygiene.

## Results

**Figure 1.**
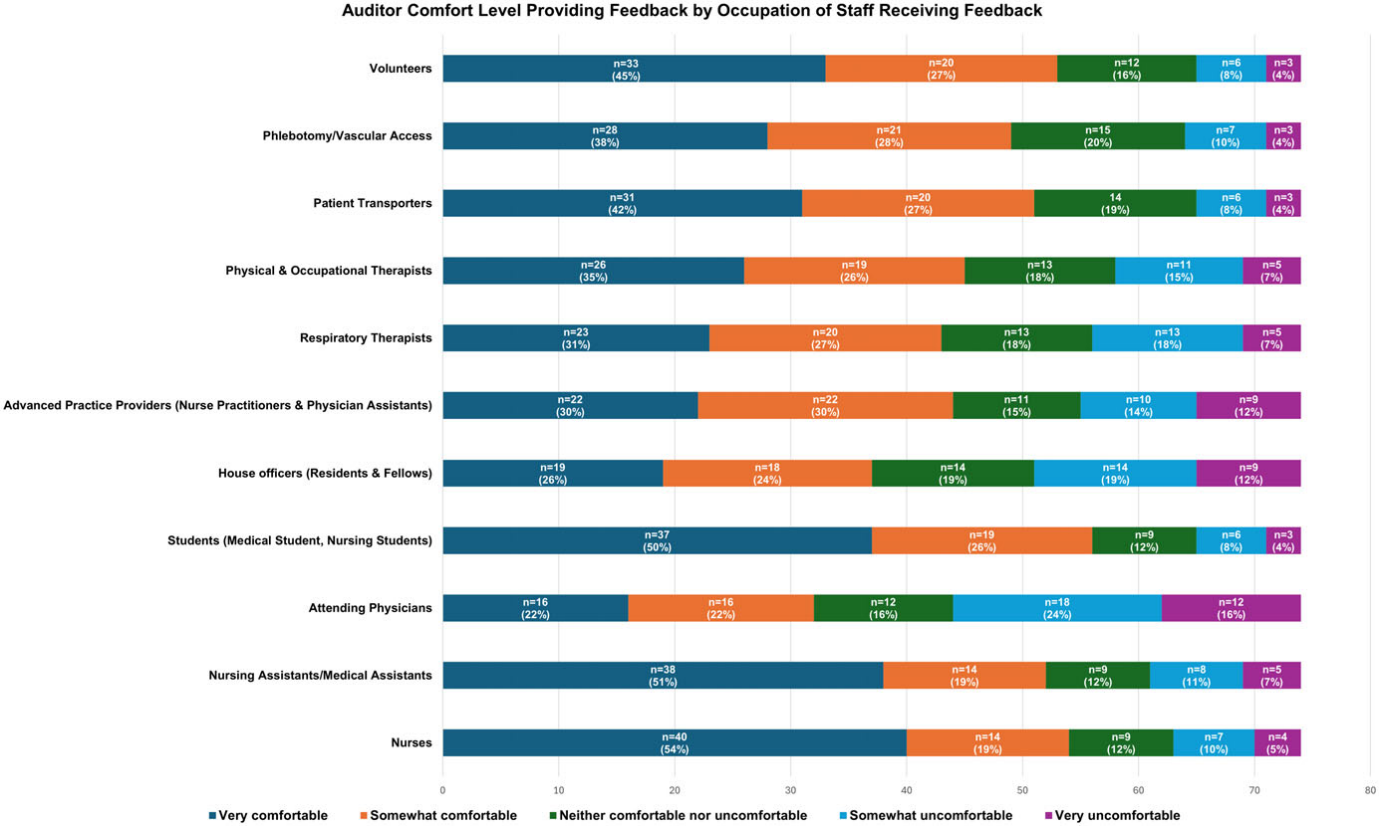
Auditor comfort level providing feedback by occupation of staff receiving feedback.

The survey was completed by 76 of 216 auditors (35%). Most auditors reported feeling “very” or “somewhat” comfortable providing feedback to staff on hand hygiene practices (n = 56, 74%); however, this varied by the occupation of the staff member receiving feedback (Figure 1 & Table 1). Auditors were most comfortable providing feedback to students with 76% of respondents (n = 58) feeling “very” or “somewhat” comfortable. This was followed by nursing staff and volunteers with 74% of respondents (n = 56) feeling “very” or “somewhat” comfortable providing feedback to both nursing staff and volunteers. Auditors were least comfortable providing feedback to attending physicians and house officers with 45% (n = 34) and 51% (n = 39) feeling “very” or “somewhat” comfortable providing feedback to house officers and attending physicians, respectively. For other occupations, auditor comfort levels ranged from 61% (n = 46) for advanced practice providers and respiratory therapists to 72% (n = 55) for nursing assistants and medical assistants. When asked about staff reactions to feedback, 47% of auditors (n = 36) reported staff react “neither negatively or positively”, 29% (n = 22) reported staff react “mostly positively”, 16% (n = 12) reported staff react “mostly negatively”, and 8% (n = 6) reported not providing feedback.

**Table 1.** Survey Responses (n = 76/216 total auditors; 35%)

**Question 1: Approximately how many times per month do you conduct hand hygiene audits?**	
More than 12 times per month	33 (43%)
Between 5 and 12 times per month	20 (26%)
Between times per month	20 (26%)
Less than one time per month	3 (4%)
**Question 2: In the past 12 months have you received education or training on how to conduct hand hygiene audits?**	
Yes	49 (64%)
No	25 (33%)
I am unsure	2 (3%)
**Question 3: How comfortable do you feel giving feedback to staff regarding hand hygiene?**	
Very comfortable	31 (41%)
Somewhat comfortable	25 (33%)
Neither comfortable nor uncomfortable	9 (12%)
Somewhat uncomfortable	6 (8%)
Very uncomfortable	5 (7%)
**How comfortable giving feedback to employees/staff from the following professions/departments?**	
*Nurses*	
Very comfortable	40 (54%)
Somewhat comfortable	14 (19%)
Neither comfortable nor uncomfortable	9 (12%)
Somewhat uncomfortable	7 (10%)
Very uncomfortable	4 (5%)
*Nursing Assistants & Medical Assistants*	
Very comfortable	38 (51%)
Somewhat comfortable	14 (19%)
Neither comfortable nor uncomfortable	9 (12%)
Somewhat uncomfortable	8 (11%)
Very uncomfortable	5 (7%)
*Attending Physicians*	
Very comfortable	16 (22%)
Somewhat comfortable	16 (22%)
Neither comfortable nor uncomfortable	12 (16%)
Somewhat uncomfortable	18 (24%)
Very uncomfortable	12 (16%)
*Students (Medical & Nursing Students)*	
Very comfortable	37 (50%)
Somewhat comfortable	19 (26%)
Neither comfortable nor uncomfortable	9 (12%)
Somewhat uncomfortable	6 (8%)
Very uncomfortable	3 (4%)
*House officers (Residents & Fellows)*	
Very comfortable	19 (26%)
Somewhat comfortable	18 (24%)
Neither comfortable nor uncomfortable	14 (19%)
Somewhat uncomfortable	14 (19%)
Very uncomfortable	9 (12%)
*Advanced Practice Providers (Nurse Practitioners & Physician Assistants)*	
Very comfortable	22 (30%)
Somewhat comfortable	22 (30%)
Neither comfortable nor uncomfortable	11 (15%)
Somewhat uncomfortable	10 (14%)
Very uncomfortable	9 (12%)
*Respiratory Therapists*	
Very comfortable	23 (31%)
Somewhat comfortable	20 (27%)
Neither comfortable nor uncomfortable	13 (18%)
Somewhat uncomfortable	13 (18%)
Very uncomfortable	5 (7%)
*Physical & Occupational Therapists*	
Very comfortable	26 (35%)
Somewhat comfortable	19 (26%)
Neither comfortable nor uncomfortable	13 (18%)
Somewhat uncomfortable	11 (15%)
Very uncomfortable	5 (7%)
*Patient Transporters*	
Very comfortable	31 (42%)
Somewhat comfortable	20 (27%)
Neither comfortable nor uncomfortable	14 (19%)
Somewhat uncomfortable	6 (8%)
Very uncomfortable	3 (4%)
*Phlebotomy/Vascular Access*	
Very comfortable	28 (38%)
Somewhat comfortable	21 (28%)
Neither comfortable nor uncomfortable	15 (20%)
Somewhat uncomfortable	7 (10%)
Very uncomfortable	3 (4%)
*Volunteers*	
Very comfortable	33 (45%)
Somewhat comfortable	20 (27%)
Neither comfortable nor uncomfortable	12 (16%)
Somewhat uncomfortable	6 (8%)
Very uncomfortable	3 (4%)
**Question 6: In general, how do staff respond when you provide them with hand hygiene feedback?**	
Mostly positively	22 (29%)
Neither positively or negatively	36 (47%)
Mostly negatively	12 (16%)
I do not provide feedback to staff	6 (8%)
**Question 7: How easy or difficult do you find the** ***Qualaris*** **platform to use?**	
Extremely easy	49 (64%)
Somewhat easy	19 (25%)
Neutral	6 (8%)
Somewhat difficult	1 (1%)
Extremely difficult	1 (1%)
**Question 8: In the past 6 months what trends have you noticed with hand hygiene compliance?**	
Significant increase in compliance	14 (18%)
Slight increase in compliance	37 (49%)
No change in compliance	18 (24%)
Slight decrease in compliance	6 (8%)
Significant decrease in compliance	1 (1%)

Most respondents (n = 49, 64%) reported receiving education/training on how to audit hand hygiene in the prior 12 months while 33% (n = 25) reported they had not received training/education and 3% (n = 2) stated they were unsure if they had received education/training in the prior 12 months. When asked about the on-line platform to record hand hygiene audits, only two participants reported finding the platform either “extremely” or “somewhat” difficult to use while 89% (n = 68) of participants reported the platform was either “somewhat” or “very” easy to use.

Free response comments were analyzed for themes and barriers to achieving hand hygiene compliance goals. The responses demonstrated a shared recognition of the importance of hand hygiene; however, the comments also identified several barriers to hand hygiene compliance. These included inconsistent provision of feedback to HCWs, challenges related to institutional culture, and infrastructure issues such as limited availability of sinks and inconvenient/infrequent placement of hand sanitizer dispensers. Staff also cited a need for increased accountability and involvement of leadership in promoting hand hygiene compliance.

## Discussion

This survey provides insights into barriers to achieving high HCW hand hygiene compliance from the perspective of hand hygiene auditors. The results also provide data that will inform future interventions aimed at improving HCW hand hygiene compliance. This survey builds on the results of prior studies and identifies auditor comfort providing feedback to HCWs and staff reactions to receiving feedback as two potential targets for interventions to improve hand hygiene compliance.^4,5^

Some studies suggest that immediate feedback during observations does not significantly improve hand hygiene compliance and the updated SHEA/IDSA/APIC guidance advises against using direct overt observation for compliance rates outside specific contexts. Despite this, monitoring hand hygiene and providing feedback remains an important metric assessed by regulatory and watchdog organizations such as The Leapfrog Group.^1,6,7^ The Leapfrog hand hygiene standards require hospitals to collect data on a prespecified number of hygiene opportunities per patient care unit each month, provide feedback to staff, and use an electronic monitoring system or direct observation methods to monitor HCW hand hygiene complaince.^7^ To accomplish this, BUH employes a combination of both direct overt and direct covert observations is used to monitor hand hygiene compliance and provide HCWs feedback.

We found most BUH auditors were comfortable providing feedback to staff regarding their hand hygiene practices; however, comfort levels varied significantly by occupation of the staff member receiving feedback. Furthermore, most auditors reported staff have positive or neutral reactions to receiving feedback on hand hygiene practices; however, approximately a quarter of auditors reported staff have mostly negative reactions to feedback or that they do not provide feedback to staff at all. The auditors’ responses suggest there may be factors that deter them from providing consistent feedback which may affect the quality and frequency of feedback provided. These results build on prior studies showing hierarchical dynamics and factors related to the institutional culture may influence auditors’ comfort in providing feedback to certain staff.^4,5,8^ This highlights the necessity for interventions that foster effective communication and feedback strategies, that empower auditors to confidently and consistently provide feedback across all occupations, and that address the perceived value of feedback among staff.^1,8,9^

This survey was subject to several limitations including a relatively small sample size of and a relatively low response rate. Further, this survey was not comprehensive and only included eight multiple choice questions and one-short answer question. Finally, the results of this survey may have limited generalizability as it was conducted at a single healthcare system.

These survey results highlight the necessity of a multi-pronged approach that combines accountability, education, infrastructure needs, cultural change, and innovative feedback methods when developing interventions focused on improving HCW hand hygiene compliance. Initiatives aimed at improving hand hygiene in healthcare settings should focus on fostering a culture of safety and accountability, encouraging openness to feedback, and engaging leadership to champion hand hygiene interventions.^1,8–10^
